# Looking beyond the audiogram in ototoxicity associated with platinum-based chemotherapy

**DOI:** 10.1007/s00280-019-04012-z

**Published:** 2019-12-21

**Authors:** David M. Baguley, Pattarawadee Prayuenyong

**Affiliations:** 1 Hearing Sciences, Division of Clinical Neuroscience, School of Medicine, University of Nottingham, Nottingham, UK; 2 NIHR Nottingham Biomedical Research Centre, Ropewalk House, 113 The Ropewalk, Nottingham, UK; 3 Nottingham University Hospitals NHS Trust, Nottingham, UK; 4 Department of Otorhinolaryngology Head and Neck Surgery, Faculty of Medicine, Prince of Songkla University, Songkhla, Thailand

**Keywords:** Ototoxicity, Platinum-based chemotherapy, Hearing loss, Audiogram

## Abstract

**Introduction:**

Ototoxicity associated with platinum-based chemotherapy is highly prevalent and can cause detrimental consequences among cancer survivors.

**Discussion:**

In this article, we highlight important aspects of the evaluation of ototoxicity with the aim to increase awareness of Oncologists in this regard. Standard pure tone audiometry alone is inadequate for this context. Comprehensive and consistent hearing tests should be implemented in a monitoring and surveillance program. High-frequency audiometry (10–16 kHz) is a sensitive tool in the detection of ototoxic hearing loss at onset. In addition to threshold audiometry, measures of speech comprehension (both in quiet and in noise) can add useful information in the evaluation of hearing in real-life situations. Not only hearing loss, but also tinnitus and imbalance are common in patients who receive platinum-based chemotherapy, and can cause debilitating effects upon quality of life in this population. Moreover, self-report measures associated with cochlear and vestibular handicaps can provide valuable information regarding the impact of ototoxicity.

**Conclusions:**

It is vital to build awareness about the variety and impact of the symptoms of ototoxicity. Comprehensive evaluation of hearing status along with self-reported impact of the cochlear and vestibular handicap should be implemented in a monitoring and surveillance program for appropriate investigation and management.

## Introduction

Platinum-based chemotherapies, such as cisplatin and carboplatin, are highly effective chemotherapeutic agents used for the treatment of a variety of life-threatening common cancers including testicular, gynaecologic, bladder, head and neck, and non-small cell lung cancer [1]. Despite potent efficacy against cancer, ototoxic effects are significantly problematic which limit usage and dosage [2]. Cisplatin has been found to be the most ototoxic agent in the platinum-based chemotherapy group with associated hearing loss [3], tinnitus [4], and imbalance [5]. Carboplatin and oxaliplatin have been demonstrated to be less ototoxic [6], though audio-vestibular issues still arise.

Ototoxicity refers to drug-related damage affecting the inner ear structures, specifically to the cochlea and the vestibular labyrinth, and their associated neural structures [3, 7]. Ototoxic effects can be characterized by cochlear dysfunction (such as hearing loss, tinnitus, or hyperacusis) or vestibular dysfunction (such as vertigo, dizziness, or imbalance) or both [7]. However, ototoxicity in the published literature usually refers to hearing disorders and both terms are used interchangeably [8].

Although hearing loss is not a fatal condition, it can have significant negative impacts on communication and health-related quality of life [9], and has been associated with dementia and cognitive impairment [10], poor mental health, and psychosocial functioning [11]. Approximately 70% of people with hearing loss had limited employment opportunities, failed to fulfill their potential at work, and felt isolated at work [12]. In children, hearing is substantially associated with speech and language development [13]; thus, hearing impairment can cause detrimental educational, vocational, and social consequences [14]. Additionally, it also has a hidden cost to society, such as reduced work productivity, if people are not offered appropriate interventions [15].

Early identification of ototoxicity might provide oncologists with an opportunity to adjust the chemotherapy regimen, either lowering the dosage or change to alternative drug, to reduce or prevent further hearing deterioration [16]. The primary aim of cancer treatment has been always to increase overall survival; however, quality of life has been increasingly documented as an important end point [17]. There is a lack of information on efficacy of ototoxicity monitoring and its cost–benefit ratio on treatment alteration [16]. There is limited information on the trade‐off between longevity and quality-of-life cancer patient is willing to make [18]; therefore, the clinical decisions in oncology clinics are made on a case-by-case basis. In a study, Enoch et al. [19] suggested that hearing and balance were ranked in top three of the most valuable senses in a general adult population and participants preferred, on average, 6.8 years of perfect health over 10 years without hearing in a time trade-off exercise. This indicates that prolonged life with reduced hearing is of diminished value.

## Discussion

Whilst there are many studies that report ototoxicity associated with platinum-based chemotherapy, the literature is characterized by small sample sizes, inadequate baseline measures, and non-standard reporting of audiometric measures. The prevalence of platinum-based ototoxicity in adults reported in the literature is approximately 50–80% [4, 20] and 60–90% in children [21, 22]. A high inter-individual variability in incidence and severity of hearing loss can potentially be explained by differences in pharmacokinetics and pharmacodynamics of certain drugs including individual susceptibility factors such as genetics [23], and other co-morbid conditions such as renal diseases [24]. Some emerging clinical translational research indicates that pre-chemotherapy patient genotyping could help in predicting cisplatin-associated ototoxicity when deciding treatment regimens [25, 26]. The severity of hearing loss associated with platinum-based chemotherapy within each individual seems to be dose-dependent and cumulative [27].

In this review, we argue that the prevalence of hearing loss associated with platinum-based chemotherapy can only be robustly determined by the consistent and appropriate use of measures of hearing in a strict pre, post, and long-term framework. Furthermore, we contend that the standard audiogram, a measure of the threshold of pure tone detection in quiet, is not a sufficient measure of real-world hearing. The use of extended high-frequency audiogram (HFA) (10–16 kHz) facilitates early detection of ototoxic hearing loss at onset. Measures of speech comprehension, both in quiet and in noise, should also be utilized. Additionally, we discuss that treating ototoxicity as a synonym for hearing loss excludes the prevalent issues of tinnitus and/or imbalance found in this population. Finally, whilst self-report measures of auditory or vestibular handicap are imperfect tools, they should be brought to bear in studies of platinum-induced ototoxicity to determine the impact of ototoxicity.

### Monitoring ototoxic hearing loss

Hearing loss caused by ototoxic medication has a relatively predictable pattern as it initially preferentially damages outer hair cells in the basal turn of the cochlea and then progresses to the apical turn [28]. Therefore, the classic characteristics of drug-induced hearing loss are bilaterally symmetrical sensorineural hearing loss that affects high frequencies, typically above 8 kHz [1], which are key components of the discrimination of speech in background noise and music perception. Cochlear damage often progresses undetected until a substantial hearing communication problem becomes apparent suggesting hearing decline in the speech range frequencies.

Conventional pure tone audiometry (PTA) remains the mainstay for the identification and categorisation hearing impairment in many ototoxicity grading systems [29]. A PTA may be all the testing that patients undergoing chemotherapy can tolerate, and this may be especially true of the paediatric population [8]. In some younger children, otoacoustic emissions may present an opportunity to assess cochlear health in an ear and frequency-specific manner [8, 14, 30]. Audiological assessments for ototoxicity may differ from standard hearing evaluation in the priority of testing frequencies and sequence of testing [30]. High-frequency audiometry (HFA) is a more sensitive tool in the early identification of ototoxic changes than the standard PTA [31, 32]. However, HFA requires specific instrumentation and additional test time, and, in practice, a change in hearing higher than 8 kHz generally does not impact the continuation of treatment regimen. Studies have revealed the ability to detect the early drug-induced cochlear damage through a limited behavioral test frequency range, called the sensitive range of ototoxicity (SRO) [33]. The SRO is a pure tone screening procedure in which a one-octave individualized range of frequencies at the high-frequency limit of hearing is monitored. The SRO is defined as the highest frequency with a threshold ≤ 100 dB followed by six lower consecutive frequencies in 1/6th-octave steps; thus, it is unique for each individual’s audiometric configuration. Testing these seven frequencies identifies approximately 90% of initial ototoxic hearing shifts [34]. The SRO procedure is both sensitive and time-efficient technique. Identifying the SRO is relatively quick while maintaining the sensitivity compared with PTA and HFA, and can be assessed using an extended high-frequency audiometer [33].

Clinically, ototoxicity is diagnosed by comparing functional status before and after the administration of ototoxic drugs; hence, baseline evaluation is essential. This prevents inaccurate diagnosis of iatrogenic ototoxic hearing loss actually caused by previous hearing impairment prior to chemotherapy treatment such as presbycusis or noise-induced hearing loss. This is particularly important in adult population as those conditions share similar audiometric results to those caused by ototoxic medications. Obtaining pre- and post-treatment hearing assessments also support basic and clinical research on drugs or interventions that can neutralize ototoxicity while not interfering with the efficacy of the anti-neoplastic capabilities of chemotherapy. Pre-existing hearing status in combination with cisplatin cumulative dose can be useful in the prediction of the degree of ototoxic hearing loss [35]. A tool to help relatively precise predictions regarding the potential reduction in hearing in advance of chemotherapy treatment would be valuable for pretreatment counselling and oncology treatment planning.

Drug-induced hearing loss is generally irreversible and occurs in a dose-related and cumulative fashion [35, 36]. Consequently, a regular monitoring program is crucial for the early detection of ototoxicity which provides useful information to minimize irreversible hearing loss as well as timely interventions. Hearing impairment after administration of platinum-based compounds can also be progressive for years after discontinuation of medication [14], which means that hearing loss may not only evident in patients who sustained ototoxicity during treatment. In addition, recent evidence shows that platinum is retained indefinitely in patients treated with cisplatin [37]. Thus, long-term surveillance is necessary because of the potential for progressive or delayed-onset hearing loss. American Speech-Language-Hearing Association (ASHA) recommends evaluations to be done in 1 and 3 months following discontinuation of ototoxic therapy [38]. Recent evidence-based guidance on ototoxicity monitoring in adolescents and young adults proposed ongoing 5 yearly audiometry [8].

The use of different criteria and grading systems for ototoxicity has made the analysis of published data challenging to interpret and has partly contributed to the variability in reporting the prevalence of drug-induced hearing loss throughout the literature [39]. Hence, uniformity of classification systems is essential to compare the results in both clinical practice and trial settings. A recent review article summarized numerous classification systems developed and used to classify cochleotoxicity using different audiometric criteria [29]. For example, the Common Terminology Criteria for Adverse Events (CTCAE) are widely accepted among the oncology research community as the standard grading scale for adverse events in clinical trials. Audiometric results are graded (1–4) according to the threshold change, number of frequencies affected, and indication for intervention. However, the CTCAE version 4 (2010) and 5 (2017) do not fully encapsulate the functional difference in difficulties experienced between a change in threshold from different baselines [29].

All of the available information emphasizes the importance of coherent and proper hearing measurement before, during, and after chemotherapy to determine the incidence and prevalence of hearing impairment associated with platinum-based chemotherapy. However, such monitoring programs are not routinely implemented [40] despite the existence of clinical guidelines [16, 38] and recommendations for ototoxicity surveillance [8].

### Threshold measures of hearing are insufficient: discrimination measures are needed

A complaint expressed by people with hearing impairment is of hearing difficulties in noisy environments [41], though, sometimes, hearing loss is not detected with routine hearing testing [42]. PTA, measuring tone detection threshold of varying frequency in quiet condition, is a poor indicator of speech recognition in noise ability, so it may not reflect dynamic real-world hearing status nor predict the handicap produced by hearing loss [43].

Speech discrimination abilities may be affected as well as detection of hearing thresholds after receiving platinum-based chemotherapy [44]. Hearing in noise testing is not commonly utilised in ototoxicity monitoring programs [16, 38], so challenges in hearing discrimination and intelligibility of speech are not routinely assessed. We propose that the evaluation of hearing perception in the presence of noise in addition to the hearing in quiet conditions will add valuable information in comprehensive hearing evaluation and help with hearing rehabilitation plan and, therefore, should be implemented in clinical practice. Examples of clinically-feasible speech-in-noise tests that are quick to administer are the Words-In-Noise (WIN) test and Quick Speech-In-Noise Test (QuickSIN) [45].

### Tinnitus

Tinnitus is a subjective perception of sound, for example, hissing, whistling or buzzing, without an external source [46], and which can lead to significant negative impacts on psychological status and quality of life [47]. Tinnitus is more prevalent in patients with hearing loss compared to normal hearing populations [48, 49]. Regarding ototoxicity, in a series of adult patients treated with cisplatin, 59% experienced tinnitus, whereas 18% had hearing loss only and 23% had both symptoms [50]. Frisina et al. [4] reported approximately 40% of testicular cancer survivors who received cisplatin complained of tinnitus which was significantly correlated with reduced hearing. On the other hand, Arora et al. [51] revealed 6 out of 57 (10.5%) patients had tinnitus irrespective of the dose of cisplatin and none of them had complained of subjective hearing loss. A recent study evaluated long-term ototoxicity in pediatric patients received platinum-based chemotherapy and/or radiotherapy reported that 66.7% of patients reported tinnitus, although they may have normal hearing detected by standard audiometry [52]. The prevalence of tinnitus associated with platinum-induced ototoxicity is unclear in the literature because of the scant research in this area, but it is likely to be underreported and underappreciated [21].

### Vestibular symptoms

Vestibular effects associated with platinum-based chemotherapy are less frequently described in the literature compared to auditory symptoms, but can be debilitating [5]. Vestibular dysfunction can trigger a deterioration of quality of life including physical impairment that interfere with driving, riding a bicycle, and other activities involving good balance, as well as psychological symptoms [53]. More than 50% of subjects with dizziness report reduced efficiency at work, 27% changed their jobs, 21% gave up work, and patients report other considerable impacts on personal and social life [54].

Ototoxic medications are generally administered systemically and, therefore, affect both ears simultaneously. Bilateral symmetrical gradual vestibular loss usually results in insidious disequilibrium, postural imbalance, or oscillopsia [55]. A compensated vestibular loss may not be recognized until the patient loses other cues from vision and somatosensory inputs such as when walking in the dark or when concomitant peripheral neuropathy is developed [5]. Moreover, there are multiple factors such as the general deconditioning of cancer patients that can make the clinical identification of vestibulotoxicity more complicated. Most patients are unlikely to have intense symptoms of imbalance; hence, subtle or suspicious symptoms of vestibular impairment should be recorded and/or undergo further investigations, for example, vertigo, dizziness, double vision, ataxia, and light-headedness [55, 56]. Vestibulotoxicity associated with platinum-based chemotherapy seems to be underinvestigated and underestimated [5]. Clinicians should be vigilant to the presenting symptoms of vestibular impairment in this patient population.

### Measurement of impact

Although testing can detect hearing loss at an early stage, the impact upon daily activities and quality of life as assessed by self-report is also important. One common limitation of the available cochleotoxicity grading systems is a lack of indication of significant clinical change of hearing linked to reduced communication function and quality of life [29]. Similar audiological characteristics in different individuals may demonstrate varying degrees of communication difficulties [57]. Self-reports of hearing difficulty generally have a higher prevalence than test measures in population studies [43], and patients may have a substantially greater hearing handicap and disability than would be expected from the results of the audiogram [44].

Self-report measures of communication difficulty should complement audiometric examinations in monitoring protocol to guide treatment plans and hearing rehabilitation as well as to gain a better understanding of the incidence and burden of ototoxicity. An example of patient-reported tools is the Scale of Chemotherapy-Induced Neurotoxicity (SCIN) [58]. Patients answer in four categories ‘not at all’, ‘a little’, ‘quite a bit’, and ‘very much’ to the questions: ‘Have you suffered from reduced hearing?’ and ‘Have you suffered from ringing in your ears?’. The impact of tinnitus and dizziness on quality of life can be evaluated using the Tinnitus Handicap Inventory (THI) [59], and the Dizziness Handicap Inventory (DHI) [60], respectively. The use of questionnaires in the paediatric population is not presently supported by validated instruments.

## Conclusions

Ototoxicity associated with platinum-based chemotherapy is a salient issue. It should be a priority to build awareness among patients and healthcare providers about the significance and variety of symptoms of ototoxicity such as reduced hearing, tinnitus, and imbalance. Comprehensive and robust baseline hearing tests within a monitoring and surveillance program should be scheduled to assess prevalence of hearing loss associated with platinum-based chemotherapy. Measures of speech-in-noise complement speech discrimination testing in quiet conditions and can add helpful information in the evaluation of real-life hearing abilities. Furthermore, the potential impact of cochlear and vestibular handicap caused by ototoxicity should be assessed by self-report measures.
